# Dynamic Control of Cell Cycle and Growth Coupling by Ecdysone, EGFR, and PI3K Signaling in *Drosophila* Histoblasts

**DOI:** 10.1371/journal.pbio.1000079

**Published:** 2009-04-07

**Authors:** Nikolay Ninov, Cristina Manjón, Enrique Martín-Blanco

**Affiliations:** Instituto de Biología Molecular de Barcelona, Consejo Superior de Investigaciones Científicas, Barcelona, Spain; University of California, San Francisco, United States of America

## Abstract

Regulation of cell proliferation has been extensively studied in cultured cell systems that are characterized by coordinated growth and cell-cycle progression and relatively uniform cell size distribution. During the development of multicellular organisms, however, growth and division can be temporally uncoupled, and the signaling pathways that regulate these growth programs are poorly understood. A good model for analyzing proliferation control in such systems is the morphogenesis of the *Drosophila* adult abdominal epidermis by histoblasts. These cells undergo a series of temporally regulated transitions during which neither cell size nor division rate is constant. The proliferation of histoblasts during metamorphosis is uniquely amenable to clonal analysis in combination with live imaging. Thereby, we show that abdominal histoblasts, which grow while in G2 arrest during larval stages, enter a proliferative stage in the pupal period that is initiated by ecdysone-dependent *string/*Cdc25 phosphatase transcription. The proliferating histoblasts have preaccumulated stores of Cyclin E, which trigger an immediate S phase onset after mitosis. These rapid cell cycles lack a G1 phase and result in a progressive reduction of cell size. Eventually, the histoblasts proceed to a stage of slower proliferation that, in contrast to the preceding, depends on epidermal growth factor receptor (EGFR) signaling for progression through the G2/M transition and on insulin receptor/PI3K-mediated signaling for growth. These results uncover the developmentally programmed changes coupling the growth and proliferation of the histoblasts that form the abdominal epidermis of *Drosophila*. Histoblasts proceed through three distinct stages: growth without division, division without growth, and growth-coupled proliferation. Our identification of the signaling pathways and cell-cycle regulators that control these programs illustrates the power of in vivo time-lapse analyses after clone induction. It sets the stage for the comprehensive understanding of the coordination of cell growth and cell-cycle progression in complex multicellular eukaryotes.

## Author Summary

A fundamental issue in biology is the question of how the rate of cell division is coupled to cell growth. Coordination of these processes has been studied extensively in cultured cell systems but to a much lesser extent in intact organisms. To study this phenomenon in a physiological setting, we developed a methodology to observe and manipulate cell division and growth in a population of *Drosophila* abdominal cells called histoblasts. The various developmental stages of histoblast morphogenesis include exit from cell-cycle arrest, initially rapid growth in the absence of cell division, and subsequent coupling of proliferation and growth. We identified several critical developmental signaling pathways (including signaling via ecdysone, the EGF receptor, and PI 3-kinase) that regulate and coordinate cell growth and division cycles during these different types of cell-cycle phenomena. We propose that the internal logic of the *Drosophila* histoblast system may serve as a basic framework for understanding not only how coordinated cell growth and division operate in a number of other developmental contexts, but also how misregulation of cell growth and division occurs in contexts such as cancer cell populations.

## Abstract 3

Integration of the ecdysone, EGF receptor, and PI 3-kinase signaling pathways determines the relative rates of growth and cell division during*Drosophila* abdominal cell morphogenesis.

## Introduction

Morphogenesis involves the coordination of a wide variety of cellular activities, including progression through the cell cycle, cell growth, and cell rearrangement. Over the last decades considerable progress, mostly in cultured cells, has been made in the identification of the crucial regulators that govern cell-cycle progression and growth. In general, cells proceed through canonical cell cycles in which S and M phases are separated by G1 and G2 phases. Passage beyond early G1 usually depends on growth factors and mitogens. Without such factors, cells halt growth and cell-cycle progression, and enter G0. When present, these factors stimulate a cascade of events culminating in the activation of G1 cyclin/Cdk complexes, which restart cell-cycle progression and lead the cells into S phase (DNA replication). The second regulated cell-cycle transition, progression from G2 into M, is also controlled by Cdk activity. Importantly, the corresponding mitotic cyclin/Cdk complexes are activated by Cdc25 phosphatases, which remove inhibitory phosphate modifications from Cdk1. For maintenance of cell size, cell-cycle progression must be accompanied by cell growth. A key regulator of cellular growth is the phosphoinositide 3-kinase (PI3K). Inhibition of the PI3K signaling pathway reduces cell, organ, and organism size (reviewed in [1]).

Cell proliferation in developing organisms involves in many cases a programmed temporal uncoupling of growth and progression through the cell cycle, with stage- and tissue-specific deviations from the canonical form. At the onset of embryogenesis, cell divisions are often extremely rapid. Well-studied examples include the syncytial cleavage cycles in *Drosophila* embryos, the cleavage stages in *Xenopus*, and the early embryonic divisions in Caenorhabditis elegans (reviewed in [2]). In these processes, the high speed of early embryonic cell-cycle progression is in part enabled by growth beforehand during oogenesis, which results in cells with abundant maternally derived stores. The presence of these stores eliminates the need for gene transcription during the initial cycles and also explains the absence of G1 and G2 phases. Maternally derived Cyclin E is thought to trigger an immediate entry into S phase after each mitosis. Moreover, high levels of maternally derived mRNAs for mitotic cyclins and Cdc25 allow a rapid onset of mitosis very soon after completion of S phase. In *Drosophila*, G1-less cell cycles continue even after cellularization, which follows the 13th syncytial S/M cycle. In these cell-division cycles, however, entry into mitosis, and thereby the length of the G2 phase, becomes controlled by *string* (the Cdc25 homolog) transcription [3]. Unconventional cell cycles without G1 are also characteristic of mouse and human embryonic stem cells (ESC) as well as some tumor cells (reviewed in [4,5]).

Rapid, growthless early cycles result in a progressive cleavage of the zygote into increasingly smaller cells. Previous growth followed by the partitioning of large cells into smaller cells is not only observed in the context of oogenesis and early embryogenesis. For instance, *Drosophila* neuroblasts, which remain quiescent during the early larval stages, initially increase in size before their size is again reduced during progression through asymmetric divisions [6].

The progenitor abdominal histoblasts in *Drosophila*, which remain quiescent during the larval stages, undergo rapid proliferation after pupation and eventually form the adult abdominal epidermis by replacing the larval epidermal cells (LECs). As shown earlier [7] and also in this work, these cells provide a highly accessible system for a detailed analysis of the temporally programmed molecular mechanisms that control the coupling of cell growth to cell proliferation. Histoblasts are specified during embryogenesis and are organized in small nests of cells surrounded by LECs. While LECs grow and endoreduplicate, histoblasts remain arrested in a G2 phase during larval stages. They also grow during this period although far less extensively than the LECs. Importantly, in contrast to the LECs, they reenter mitotic cell-division cycles at the onset of metamorphosis. Early, from 0 h to 8 h after puparium formation (APF), they undergo three very rapid and synchronous growthless divisions (around 2.5 h each). Later, from 8 h to 36 h APF, the length of their cell cycle increases progressively (up to 8 h), and they undergo interphase growth while keeping their overall size constant [8,9]. After invading and replacing the larval epithelium, histoblasts acquire epidermal and neural fates and terminally differentiate. How histoblast cell divisions and growth are genetically regulated and interconnected is essentially unknown.

In this paper, we identify the signaling pathways and their target regulators that, following pupation, control cell-cycle reentry, cell-cycle speed, and growth during histoblast proliferation. Our results are derived from experiments exploiting a novel and powerful combination of clonal analyses and in vivo visualization. We find that during larval stages, arrested histoblasts accumulate cellular mass in a process dependent on the insulin receptor/PI3K pathway (Stage 0—before cell-cycle entry). Thereafter, ecdysone-dependent *string* transcription triggers exit from the quiescent G2 state at the onset of metamorphosis. As a result of the accumulation of the G1/S regulator Cyclin E during larval stages, the initial cell cycles are G1-less and very fast (Proliferation Stage 1). Moreover, cell growth does not keep up with progression through these rapid cell cycles. Finally, upon depletion of stored Cyclin E, histoblasts proceed into a stage of slower proliferation (Proliferation Stage 2) in which G1 is restored. In contrast to the previous stage, cell proliferation depends on mitogenic and growth factor signaling. The epidermal growth factor receptor (EGFR) pathway is required for the G2/M transition, and the insulin receptor/PI3K pathway for cell growth. Analogous regulation might occur in other proliferative tissues and perhaps in tumors as well.

## Results

### Histoblast Exit from Cell-Cycle Arrest Relies on *string* Transcription Stimulated by Ecdysone

During larval periods, histoblasts remain arrested in the G2 phase of the cell cycle. Although they derive from cells born in embryonic mitosis 16 and are initially in G1 phase, at some point they transit S and arrest in G2 (Stage 0); i.e., they express Cyclin A, a marker of G2 ([10] and unpublished data) and are able to undergo mitotic recombination, which does not occur in G1-arrested cells [11].

**Figure 1:**
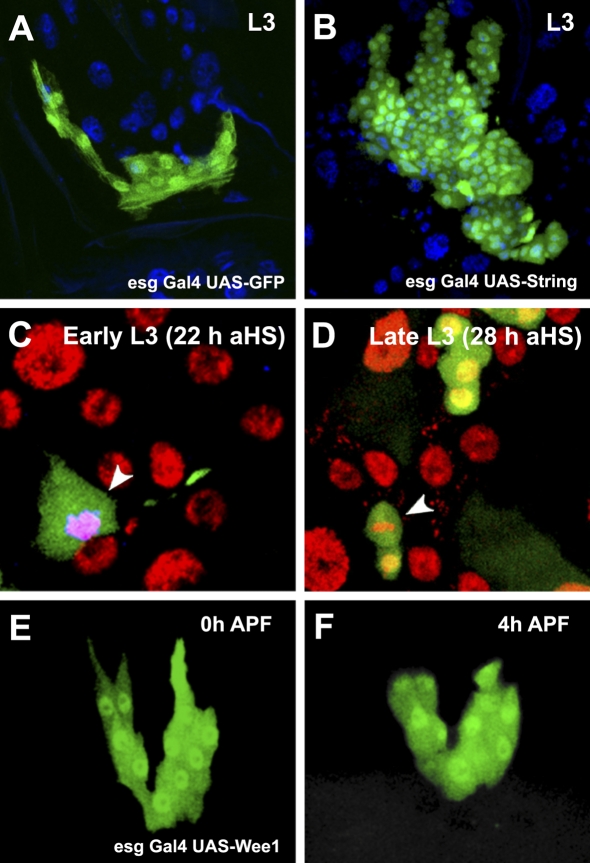
String Is Both Necessary and Sufficient for the Initiation of Histoblast Proliferation (A and B) Wild-type (A)and UAS-String–expressing (B) third instar larva (L3) ventral histoblast nests labeled with GFP. Misexpression of String induced premature proliferation of histoblasts in the larva. (C and D) UAS-String overexpressing FLP-OUT/FRT clones at 22 h (C) and 28 h (D) after heat shock (aHS) in third instar larvae (His2RFP [red] and GFP [green]). String/GFP-positive cells autonomously enter mitosis (pHis3 staining; blue) and generate multicellular clones. (E and F) Snapshots from a time-lapse movie of a UAS-Wee1–expressing ventral histoblast nests at 0 h (E) and 4 h APF (F). The misexpression of Wee1 delays the onset of histoblast proliferation, and cell numbers do not change.

At metamorphosis, histoblasts initiate a period of rapid cell divisions [9]. In eukaryotes, the transition from G2 to M is controlled by the Cdc25 tyrosine phosphatase [12] and in *Drosophila* embryos, cells homozygous for *string* arrest in G2. String overexpression triggers cell-cycle progression in embryonic and imaginal cells previously arrested in G2 [3,13,14], but not in G1-arrested cells [15]. Accordingly, the overexpression of String, but not Cyclin A, Cyclin B, or Cdk1, in histoblasts triggered their premature hyperproliferation in larval stages (compare Figure 1A with 1B; unpublished data—see Discussion). Temporally controlled overexpression of String in clones during larval stages also led to autonomous entry into M (Figure 1C and 1D). Together, these experiments confirm that histoblasts are arrested in G2. To directly test whether String was required for histoblast reentry into mitotic cell-division cycles, we overexpressed Wee-1, a tyrosine kinase that phosphorylates and inactivates Cdk1, thereby exerting a dominant-negative effect over String function [16,17]. The overexpression of Wee-1 led to cell-cycle arrest. Histoblasts were not able to reenter the cell cycle in time, and remained arrested up to 5 h APF (compare Figure 1E and 1F).

**Figure 2:**
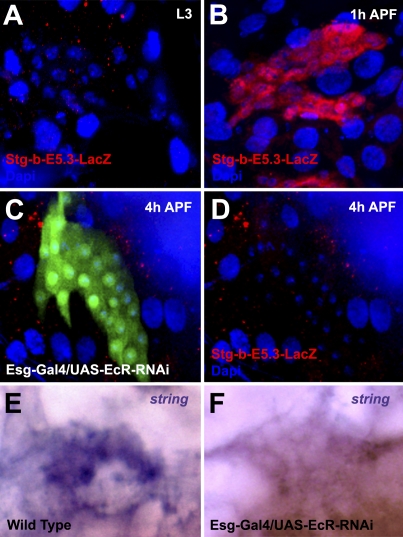
Ecdysone Signaling Is Necessary for *string* Expression in Histoblasts (A) String (String-LacZ; red) is not expressed in histoblasts during larval stages. L3, third instar larva. (B) During the early cell cycles (1–2 h APF), String-LacZ becomes strongly up-regulated in proliferating histoblasts. (C and D) Esg-Gal4, UAS-GFP; UAS-EcR-RNAi/String-LacZ pupae at 4 h APF (anterior dorsal nest). The expression of EcR-RNAi in histoblasts inhibits the expression of String-LacZ and the onset of histoblast proliferation. (E and F) In situ hybridization of a *string* probe on a wild-type (E) or Esg-Gal4, UAS-GFP; UAS-EcR-RNAi/+ (F) ventral histoblast nest at 4 h APF.

The onset of histoblast proliferation (1–2 h APF) [7] follows the ecdysone hormonal pulse that reaches its maximum at 0 h APF and promotes the larval to pupal transition [18,19]. Ecdysone is necessary to trigger histoblast proliferation [7], but experimental up-regulation of *string* transcription in larval stages (Figure 1B, 1C, and 1D) bypasses the requirement for larval–pupal ecdysone. Indeed, coexpression of EcR-RNAi, which blocks the exit of histoblasts from G2 arrest [7], does not prevent ectopic String-promoted histoblast proliferation in larval periods (unpublished data). To characterize the dynamics of *string* transcription, we analyzed the expression of a *string* histoblast-specific reporter [20]. We found that the String-b-E5.3 element is activated at the onset of proliferation in histoblasts (from 1 h APF), and is not expressed in larval stages (compare Figure 2A and 2B). After expression of an EcR-RNAi transgene in histoblasts, the early expression of the String-b-E5.3 element was abolished (Figure 2C and 2D). Consistently, the overexpression of EcR-RNAi also inhibited *string* transcript expression (monitored by in situ hybridization) (compare Figure 2E and 2F). Altogether these results indicate that Ecdysone signaling is required for *string* transcription, which triggers histoblast exit from G2 arrest at the onset of metamorphosis.

### The Early Cell Cycles of Histoblasts (Proliferation Stage 1) Proceed without G1 as a Result of the Accumulation of Cyclin E during Larval Stages

Time-lapse analysis showed that initial histoblast divisions within segments are metasynchronous, lasting about 2.5 h (Proliferation Stage 1), and progress without interphase cell growth (Figure S1A and Movie S1). After these first cycles (three prepupal cycles: 1–8 h APF), cell-division synchrony decreased, and cells divided in random clusters [7]. During Proliferation Stage 2 (16–24 h APF), the cell-cycle doubling time increased progressively up to 8 h, and cells grew between cell divisions (Figure S1B and Movie S2). Cell sorting (FACS analysis) showed that during the fast prepupal Stage 1, histoblasts skipped or underwent a very quick G1, whereas G1 was recovered during the slow pupal Stage 2 [7].

**Figure 3:**
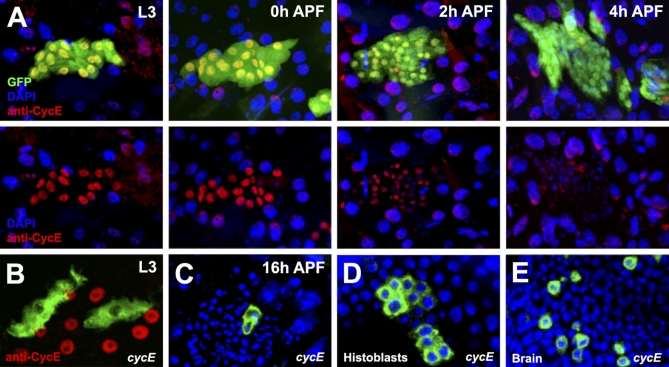
Cyclin E Accumulates in Histoblasts in the Larva and Facilitates the Early Cell Cycles (A) Histoblasts from larval and early pupal stages were stained with DAPI and Cyclin E and specifically labeled using GFP. Cyclin E levels are high in histoblasts in the third instar larva (L3) and before the onset of proliferation at 0 h APF. During the first two cycles (2 h and 4 h APF), the stored Cyclin E decreases to barely detectable levels. (B) *cycE* mutant clones were induced in the embryo and positively marked with GFP using MARCM. Staining in the third instar larva shows that mutant cells (green) fail to accumulate Cyclin E. Such cells arrest after only one division and form two cell clones (C). (D) *cycE* mutant clones were generated after the first cell division in pupal stages and were positively marked with GFP using MARCM. At late stages (30 h APF), clones are composed of up to six cells, suggesting that each mutant *cycE* cell has divided at least two or three times. In contrast, mutant cells in other tissues like the brain (E) and the wing (unpublished data) are arrested and form mostly one- or two-cell clones.

In *Drosophila*, the critical rate-limiting factor for G1 to S transition is Cyclin E. Cyclin E shows cyclic expression and accumulates only during late G1, where it associates with Cdk2 and promotes entry into S. Cells from mutants for *cycE* or *cdk2* become arrested in G1, whereas the overexpression of CycE shortens the G1 phase [16,21]. Strikingly, we detected high levels of Cyclin E by immunohistochemistry in third instar larvae before histoblasts initiate divisions (Figure 3A). We also found that *cycE* loss-of-function clones generated in the embryo, which do not accumulate CycE in larval periods (Figure 3B), promptly arrest in Proliferation Stage 1 (Figure 3C).

Interestingly, stored Cyclin E declined progressively during the first cell cycles (Stage 1) and was depleted by the third prepupal cycle at 4 h APF in accordance with a decrease in the synchrony and speed of cell division. This observation suggests that the ability of histoblasts to undergo fast G1-less divisions may be a consequence of the accumulation of Cyclin E (and possibly other G1 regulators) during larval stages. This stored Cyclin E would be enough to carry histoblasts through G1 without de novo synthesis. To test this hypothesis, we generated *cycE* loss-of-function clones just after the first prepupal cell division. At late developmental stages (24–30 h APF), we detected histoblast clones composed, on average, of four to six *cycE* cells, indicating that each mutant cell had divided at least twice without new Cyclin E transcription (Figure 3D). In contrast, *cycE* control clones in pupal brains and wings showed impaired proliferation and were formed mostly of one or two cells (Figure 3E and unpublished data). These results show that histoblasts rely on the pool of stored Cyclin E protein for G1 transition in the prepupal Stage 1. Indeed, clonal overexpression of Dacapo, a specific inhibitor of Cyclin E, results in cell-cycle arrest during this stage (unpublished data).

### EGFR Signaling Is Involved in G2/M Progression during Proliferation Stage 2

To determine which signaling pathways could be instrumental in determining the duration and the speed at which histoblasts will divide in pupal periods, we induced MARCM loss-of-function clones for receptors or downstream effectors of the EGF, insulin/PI3K, JAK/STAT, FGF, Hh, Wg, Dpp, JNK, and PVF pathways (unpublished data). Cell numbers in these clones were compared to wild-type ones at late pupal Stage 2 (24–28 h APF) (see Materials and Methods). On average, each wild-type clone contained 37 ± 5 cells resulting from five cell divisions.

**Figure 4:**
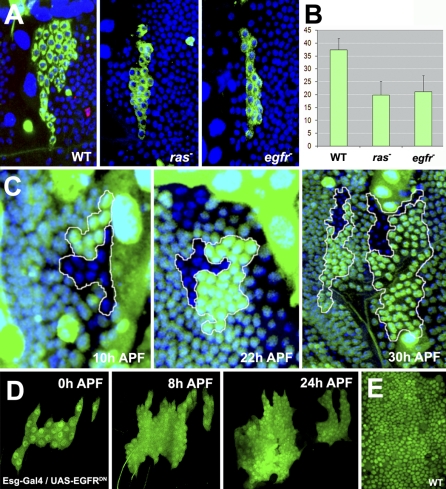
EGFR/Ras Signaling Is Necessary for the Second Stage of Histoblasts Proliferation (A) Representative clones induced in the blastoderm by MARCM and analyzed in the interval 24–28 h APF in anterior dorsal nests. Wild-type (WT) clones have more cells than homozygous *ras* or *egfr* mutant clones. A mitotic figure labeled with phospho-histone 3 (pHis3; red) could be observed outside of the clone in the WT panel. (B) Quantification of cell numbers per clone for the three genotypes (WT = 37 [*n* = 17], *ras* = 20 [*n* = 19], *egfr* = 21 [*n* = 9]). Error bars represent the standard deviation based on the number of cells per clone. (C) Twin clonal analysis (ventral nest) of *egfr* clones induced in the larva shows that at 10 h APF, mutant cells (marked by absence of GFP) have the same proliferation rate as twin wild-type cells (bright green). At 2 2 h (ventral nest) and 30 h APF (dorsal nest), the wild-type twins outnumber *egfr* mutant cells. (D) Snapshots from a time-lapse analysis of a dorsal nest expressing a dominant-negative EGFR (UAS-EGFR^DN^) using the permanent Esg-Gal4 driver (Movie S3). The expression of EGFR^DN^ does not affect histoblast proliferation in early cell cycles, and histoblasts triplicate by 8 h APF. Anterior and posterior dorsal nests expressing EGFR^DN^ have stopped proliferation at 24 h APF (E). The anterior and posterior dorsal nests failed to fuse and contain fewer cells than wild-type nests at this stage of development.

It has been shown that *EGFR* hypomorphic alleles present abdominal defects and a reduced number of histoblasts [22]. Strikingly, clones of *egfr* or *ras* showed a reduced number of cells: 20 ± 5 and 22 ± 6, respectively (Figure 4A and 4B). To directly compare proliferation rates of *egfr* and wild-type cells, we induced twin-spot clones in larval stages. Mutant clones compared to wild-type twins showed no difference in cell numbers during Stage 1 (10 h APF) but a strong reduction at late stages (22–30 h APF) (Figure 4C). This suggested that EGFR signaling is specifically involved in Stage 2 of histoblast proliferation and, accordingly, movies following *egfr* clones rarely display mitotic figures during this stage (unpublished data).

**Figure 5:**
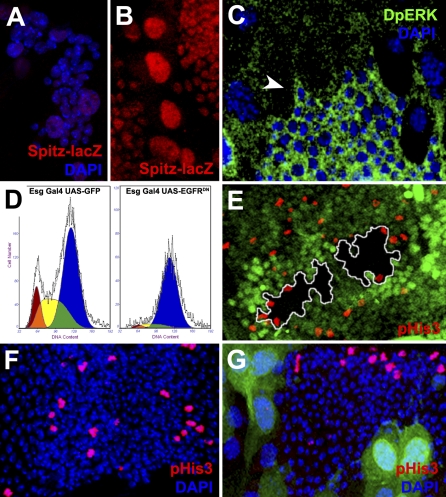
EGFR/Ras Is Active in Histoblasts and Promotes G2/M Progression (A and B) *spitz* (Spitz-LacZ; red) is not expressed at 1 h APF (A) but is strongly up-regulated in both histoblasts and LECs at 24 h APF (B). (C) DpERK (green) is expressed in histoblasts (arrowhead) and LECs (dorsal nest: 22 h APF). (D) Expression of EGFR^DN^ using the permanent Esg-Gal4 driver blocks histoblasts at the G2 phase of the cell cycle (FACS analysis: 22 h APF)). In wild-type conditions (left), 12.5% of histoblasts are found in G1 (red), 30% in S (yellow), and 58% in G2 (blue). After EGFR^DN^ overexpression (right), histoblasts become arrested in G2 (89%) with only 2.5% found in G1 and 8.5% in S. (E) *spitz* histoblast clones (black; absence of GFP marker) stained with phospho-histone 3 (pHis3; red) to visualize cells in mitosis. Mutant histoblasts do not enter in M except at positions adjacent to wild-type Spitz-expressing cells. (F) A wild-type dorsal nest stained with PH3. Cells in mitosis are randomly present across the nest. (G) The overexpression of UAS-Argos in LECs blocks mitosis in adjacent histoblasts at the nest periphery, which are closest to the Argos-expressing LECs. The nest is smaller compared to wild-type controls (F).

Potential EGFR perdurance from larval stages in twin clones, however, could mask potential early prepupal Stage 1 requirements. To reject this possibility, we directly inhibited EGFR activity in both Stages 1 and 2 by expressing an EGFR dominant-negative construct (EGFR^DN^) using a permanent Esg-Gal4 driver (see Materials and Methods). The expression of EGFR^DN^ did not affect early fast Stage 1 divisions but resulted in a complete arrest of proliferation at around 18 h APF (after four to five cell cycles) (compare Figure 4D and 4E; Movie S3). Remarkably, FACS analysis showed that histoblasts overexpressing EGFR^DN^ became arrested in G2 at the end of the prepupal Stage 1 (Figure 5D).

The observed reduction in cell numbers in the absence of EGFR signaling could be due to increased cell death. To rule out this option, we carefully analyzed several movies depicting the growth of *egfr* clones during Stages 1 and 2 and found that only 5% of the cells undergo delamination, a figure not significantly different from that obtained for wild-type clones. Further, activated Caspase-3 staining in fixed samples hardly detected any dying histoblasts in *egfr* (or wild-type) clones, whereas dying LECs were easily identified (unpublished data).

Cell division arrest at late pupal proliferation Stage 2 was also observed in loss-of-function conditions for genes downstream of EGFR. We found that histoblasts mutant for the Ras-GEF, *son of sevenless* (*sos*), displayed normal proliferation rates during prepupal cycles (Figure S2A and Movie S4). As expected, twin clones for *sos* showed reduced cell numbers at late Stage 2 (24 h APF) (Figure S2B).

Altogether, these data indicate that histoblast prepupal fast divisions are insensitive to EGFR activity, which instead supports proliferation at Stage 2. This function correlates with the up-regulation of the EGFR-secreted ligand Spitz in both histoblasts and LECs of late pupae (Spitz-LacZ [23]) (compare Figure 5A and 5B) and with the hyperactivation of the EGFR/Ras signaling (detected by elevated levels of phospho-ERK staining, Figure 5C) in histoblasts. *spitz* clones in histoblasts resulted in suppression of M (absence of phospho-H3 expression) except at the periphery of the clones where M was nonautonomously rescued by adjacent wild-type Spitz-expressing cells (Figure 5E). Further, overexpression of the secreted Spitz antagonist Argos in LECs resulted in a nonautonomous suppression of M on adjacent histoblasts (Figure 5F and 5G). EGFR signaling thus responds to the mitogenic signal of Spitz, supporting G2 to M transition and sustaining cell-cycle progression in late pupal Stage 2.

### Insulin/PI3K Signaling Is Necessary for Histoblast Growth in the Late Proliferation Stage 2

Histoblast size is under strict temporal control. First, histoblasts grow without division around 60-fold during larval stages (Stage 0) ([9] and Figure S3A and S3B). They then decrease in size during the early prepupal Stage 1 divisions that lack G1. Finally, after shifting to a slow cell cycle (Stage 2), they maintain a constant cell size (Figure S1).

The size of histoblasts responds to alterations in the level of the insulin/PI3K signaling cascade. Indeed, overexpressing Dp110 (the catalytic subunit of PI3K) or the PI3K antagonist PTEN (a lipid phosphatase) using the histoblast-specific Esg-Gal4 driver led to an increase or decrease in cell size, respectively, whereas overexpression of Myc, an independent effector of cell growth unrelated to insulin/PI3K signaling, did not affect it (unpublished data).

**Figure 6:**
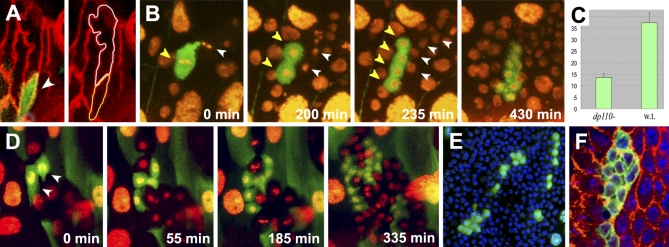
PI3K Signaling Is Required for Histoblasts Growth and Proliferation (A) A *dp110* (PI3K) cell (GFP [green]; arrowhead) induced at blastoderm using MARCM and examined at 0 h APF. The size of the mutant cell (outlined in yellow, right) is approximately 40% of its neighbor wild-type cell (outlined in white), indicating a requirement for PI3K for cell growth during larval stages. Cell membranes were stained with an anti-Dlg antibody (red). (B) Snapshots from a time-lapse movie (Movie S5) showing a single-cell *dp110* clone (GFP; yellow arrowheads) induced at blastoderm using MARCM. All cells are labeled with His2-YFP. The mutant cell enters mitosis at the same time as its wild-type neighbor (white arrowheads) and proceeds to a second division at 200 min in synchrony with the entry in cycle of the two daughter cells from the wild-type neighbor (note the condensed mitotic chromatin; His2-YFP). Overall, the *dp110* cell divides three times in 430 min and generates a clone of eight cells, as do its wild-type neighbors, indicating a normal division rate. (C) Quantification of cell numbers of wild-type (*n* = 17) and *dp110* clones (*n* = 18) induced in the embryo and examined 26 h APF. On average, *dp110* clones are composed of 14 cells, a strong reduction compared to wild-type (w.t.) clones. (D) A two-cell FLP-OUT/FRT UAS-PTEN clone generated in an early third instar larva marked with GFP. The clone was followed by time-lapse photography (Movie S7) during the early stage of proliferation (anterior dorsal nest: 0 h APF). PTEN-expressing cells undergo the first three rounds of division and generate a clone of 16 cells in 335 min, as do wild-type histoblasts (His2-YFP). (E) FLP-OUT/FRT UAS-PTEN clones analyzed at 25 h APF (ventral nest) in fixed preparation. These clones are composed of few and scattered cells. (F) *dp110* clones stained with anti-Dlg (red) and DAPI (blue) at 28 h APF. The cells of the clone show reduced size compared to wild-type cells.

To understand how cell cycle and growth were regulated during the distinct proliferation stages, we generated *dp110* (PI3K) mutant cell clones. Induction of MARCM *dp110* clones in the embryonic blastoderm prevented histoblast growth during larval stages (Stage 0) and, as a result, the *dp110* mutant histoblasts were one-third to one-half the size of wild-type cells at 0 h APF (Figure 6A). Strikingly, this reduction in size did not affect cell-cycle entry or early fast Stage 1 divisions. *dp110* mutant histoblasts proceeded through the first three cell cycles (Stage 1) at a normal speed (Figure 6B and Movie S5). The absence of Dp110, however, resulted in cell-cycle arrest or very slow histoblast proliferation during Stage 2 (Movie S6), leading to a reduction in the number of cells per clone (14 cells on average) (Figure 6C). Histoblasts from *dp110* mutant clones (18–26 h APF) did not grow and became smaller than their neighbors (Figure 6F). Similar results were obtained for MARCM clones of the *Drosophila* insulin receptor *chico* (unpublished data).

The observed late arrest of *dp110* mutant histoblasts at Stage 2 could be a consequence of their failure to grow during larval stages. To test this possibility, we inhibited PI3K signaling by overexpressing PTEN at different times in third instar larvae. Flip-out clones induced in early third instar larvae (monitored by time-lapse 24–30 h after induction) showed normal fast division rates at Stage 1 (Figure 6D and Movie S7). However, clones induced in wandering third instar larvae, once histoblasts reached their final size, showed reduced cell numbers at Stage 2 (12–14 cells) (Figure 6E).

Given the known role of PI3K signaling in cell survival, the small number of cells in *dp110* clones might be a consequence of increased cell death. To rule out this possibility, we first quantified cell divisions of *dp110* mutant cells in comparison to wild type in time-lapse movies and found that *dp110* cells divide more slowly (as an average, 21% of *dp110* cells did not divide, 52% divided only once, and 21% divided two times, whereas all wild-type cells divided, 21% once and 79% twice, in the recorded 12-h period). No delaminating/dying cells were ever observed in these movies of *dp110* clones. Further, no activated Caspase 3 staining was detected during late Stage 2 in *dp110* or PTEN overexpressing clones (unpublished data), indicating that manipulation of either PI3K or PTEN expression does not result in cell death but does affect histoblast proliferation.

Altogether, these analyses indicate that PI3K signaling is not required for early histoblast divisions but is essential during the late cell cycles for interphase growth.

## Discussion

**Figure 7:**
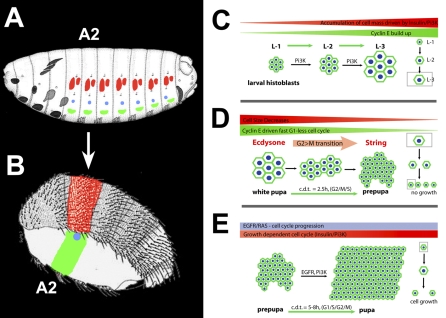
A Model of Histoblasts Cell-Cycle Progression (A) Four histoblast nests pairs are specified in each hemisegment of the embryo: Anterior dorsal nest composed of around 18 cells and posterior dorsal nest composed of five cells (red), spiracular nest (blue) formed by three cells, and a ventral nest composed of 14 cells (green). (B) During metamorphosis, histoblast nests develop to form the different structures that compose the abdominal adult epidermis, tergites and intersegmental membranes (red), spiracle (blue), and pleurites and sternites (green). The second abdominal segment (A2) is highlighted. (C) During larval stages, histoblasts arrested in G2 and grow in response to PI3K activity (Stage 0). (D) At the onset of metamorphosis, a hormonal input mediated by ecdysone is required for the expression of String, which promotes G2 arrest relief and reentry in the cell cycle. As a consequence, histoblasts undergo several fast synchronous G1-less cell cycles utilizing a stored pool of G1 regulators, including Cyclin E. During these divisions, histoblasts cleave into smaller cells, not undergoing interphase growth (Stage 1). (E) Subsequent to these cell cycles, division synchrony is lost, the cell cycle slows down with the restoration of a G1 phase, and histoblasts keep their size constant by growing between cycles (Stage 2). These late divisions are coupled to epithelial expansion and replacement of LECs. EGFR signaling triggered by the ligand Spitz is essential for progression of the cell cycle, and in the absence of EGFR signaling, histoblasts arrest in G2. Growth at this stage is mediated by insulin/PI3K signaling.

Morphogenesis of the adult abdominal epidermis depends on the finely controlled coordination of histoblast growth and cell-cycle progression. This control is mediated by the Ecdysone, EGFR, and PI3K signaling pathways modulating the activity of cell-cycle regulators (Figure 7).

### Exit from G2 Arrest

The abdominal histoblasts are arrested in G2 during the three larval instars (Stage 0) [7,10,11]. Although cell-cycle arrest in G2 is less common than in G1, there are well-known precedents in other cell types and organisms [24–26]. Our observations indicate that at the onset of metamorphosis, histoblasts exit from G2 arrest by triggering *string* transcription (Figure 2). Similar roles for *string* have been described for the cell cycle in the precellular embryo after the depletion of maternal String [3,13] and for progression of noncommitted cells through an additional cell cycle in response to EGFR activation after passage of the morphogenetic furrow (second mitotic wave) in the *Drosophila* eye imaginal disc [27,28].

Histoblast proliferation at metamorphosis depends upon an ecdysone hormonal input [7]. Here, we demonstrate that the activation of *string* transcription in histoblasts is also ecdysone signaling dependent (directly or indirectly). Thus, the ecdysone pulse at the larval–pupal transition appears to be necessary for the activation of the cell-cycle machinery that promotes exit from G2 arrest.

Previous studies reported occasional divisions of histoblasts after transient heat shock–driven expression of String in third instar larvae [10]. Consistent with these studies, we found that ectopic overexpression of String during the larval periods was sufficient to induce premature proliferation of histoblasts (Figure 1). Moreover, constitutive overexpression induced tumor-like overgrowth, apparently overriding dependence on extrinsic mitogenic signals (unpublished data). Likewise, Cdc25 overexpression has been reported in a significant number of human cancers in which deregulation of the cell cycle presumably leads to genomic instability and progression of the disease (reviewed in [29]).

### Early Fast Cell Divisions

Upon exit G2 arrest, histoblasts undergo a series of synchronous rapid cell cycles lacking a G1 phase. During these divisions, cell growth does not keep up with cell-cycle progression, resulting in a progressive reduction in cell size (S/G2/M) (Stage 1). They are analogous to the three postblastoderm divisions of the embryo, which also lack a G1, do not show substantial growth, and are roughly of comparable duration [3,13]. Similar to these cell cycles [30], the rapid early divisions of histoblasts depend on stored G1 regulators (Cyclin E) and bypass growth requirements. Other rapid G1-less early embryonic divisions in both vertebrates and invertebrates are transient, depend on Cyclin E inherited from the oocyte, bypass any growth requirement, and are insensitive to mitogens [31,32]. Interestingly, fast proliferating embryonic stem cells (ESCs) also show sustained expression of Cyclin E, absence of a cell-size checkpoint, and insensitivity to extracellular stimuli promoting cell differentiation [4]. Furthermore, hyperactivation of Cyclin E is also associated with proliferation in tumors, which is relatively independent of growth factors or differentiation stimuli [33].

Remarkably, the overexpression of Cyclin E in histoblasts failed to promote further fast cycles in Stage 1 or to increase the rate of proliferation in Stage 2 (unpublished data), suggesting that the stored Cyclin E is just permissive for fast cell cycling and must act together with additional factors that may also accumulate in larval stages. Two alternative mechanisms could account for the rapid transit into S phase. First, during Stage 1, PI3K signaling, and hence growth, could be separately repressed, e.g., in response to ecdysone. Second, above-threshold levels of G1 regulators could override growth dependence.

After the rapid early cycles, the speed of histoblast proliferation progressively decreases (Stage 2). This slowdown occurs upon depletion of stored Cyclin E. As a consequence, the histoblast cell cycle incorporates an extended G1 phase, and expression of cell-cycle regulators becomes essential. This transition is analogous to the developmental onset of G1 at cycle 17 during *Drosophila* embryogenesis [3,13]. Changes in cell-cycle speed also occur during early embryogenesis in *Xenopus* and mouse [34,35] and during late vertebrate development, as in the progressive lengthening of the cell cycle during stem cell migration [36] or during corticogenesis [37]. In most of these cases, as in histoblasts, the duration of the cell cycle mainly correlates with the length of the G1 phase.

### Sustaining Proliferation and Implementing Cell Growth during Late Stages

Histoblast proliferation does not stop upon depletion of the stored cell-cycle regulators. At least four additional divisions (Stage 2) are necessary to generate the cells of the adult abdomen. These divisions are stochastic and characterized by conventional (G1/S/G2/M) cell cycles with a doubling time of 5 to 8 h. The dividing Stage 2 histoblasts receive mitogenic input and activate growth regulatory controls. EGFR and PI3K signaling, respectively, mediate these processes.

In *Drosophila*, EGFR signaling has been shown to be involved in proliferation control during the brain expansion and in the developing eye [27,28,38,39]. Previous reports have also suggested that histoblast divisions depend on EGFR signaling [22]. We demonstrate that EGFR signaling is required for the G2/M transition during Stage 2 (Figure 5). Although mitogenic signaling by epidermal growth factor (EGF) is generally thought to control progression through the G1 phase, EGFR signaling is also required for progression through G2 in several processes in both *Drosophila* and mammals [27,28,40]. How EGFR stimulates the G2/M transition in histoblasts is not yet entirely clear, but potential targets include either positive regulators of Cdk1 activity such as String and the mitotic cyclins A and B or negative regulators such as the Wee1/Myt1 kinases.

Spitz, a diffusible EGFR ligand (reviewed in [41]), whose expression increases from 5 h APF onwards in both histoblasts and LECs, is required for EGFR activation. Indeed, wild-type histoblasts nonautonomously rescue adjacent *spitz* mutant cells, and overexpression of Argos (a Spitz antagonist) in LECs suppresses divisions in adjacent histoblasts. We previously showed that the death of LECs is spatially and temporally coordinated with the expansion of the histoblast population [7]. A paracrine effect of Spitz secreted by LECs might be part of this feedback regulation. LEC death would reduce available Spitz levels and thereby adjust histoblast proliferation.

Interphase growth is in great part responsible for the expansion of histoblast nests, and we found that insulin and PI3K signaling are necessary for both cell growth and cell-cycle progression during the late Stage 2 divisions. Histoblasts mutant for either *dp110* or *chico*, or histoblasts that overexpress PTEN, arrest at the end of the early fast cell cycles (Stage 1). Compatible with these findings, we have identified a dynamic regulation of several components of the insulin/PI3K pathway such as *chico* or *sgg* (GSK3) by transcriptomic analysis during the transition between Stages 1 and 2 (M. I. Grande and E. Martin-Blanco, unpublished data). In accordance with these results, PI3K signaling is also essential in the control of conventional cell cycles in imaginal discs, affecting both growth and proliferation [42]. Further, in zebrafish, inhibition of insulin function results in impaired embryonic growth, arrested cell divisions, and increased lethality [43]. Similarly, in mammals, PI3K promotes cell division and directs growth of postmitotic cells [44,45].

In summary, the coupling of cell growth and cell-cycle progression during *Drosophila* abdominal morphogenesis proceeds through a series of developmentally programmed stages. We found that the sequential and coordinated activities of extrinsic hormonal, mitogenic, and growth signals, respectively mediated by the ecdysone receptor, EGFR, and PI3K, regulate histoblast numbers and size. The combination of live observation and clonal analysis and the identification of the elements involved in regulating the distinct stages should allow further progress towards a molecular understanding of the developmental mechanisms that control cell proliferation in histoblasts, and the modeling of related clinically relevant processes.

## Materials and Methods

### Fly stocks.

Fly stocks were maintained on standard culture media. Crosses were performed at 25 °C. Expression of UAS constructs was conducted at 29 °C.

Hs-FLP Ay+Gal4 UAS-GFP H2YFP (*hsp70-flp; Act FRT y^+^ FRT Gal4, UAS-GFP/CyO; H2YFP/TM2*); Hs-FLP Ay+Gal4 UAS-GFP H2RFP (*hsp70-flp; Act FRT y^+^ FRT Gal4, UAS-GFP/CyO; H2RFP/TM2*); Esg-Gal4 UAS-GFP (*y, w; NP5130, UAS-GFP, UAS-nGFP, UAS-lacZ/CyO*) (NIG-FLY Stock Center); Esg-Gal4 Ay+Gal4 UAS-GFP UAS-FLP (*y, w; NP5130, Act FRT y^+^ FRT Gal4, UAS-GFP/CyO; UAS-FLP/TM6B*); UAS-EcR-RNAi (*UAS-EcR-AB RNAi*) [46]; UAS-String (*UAS-string.N4*) (Bloomington Stock Center 4778); UAS-PTEN (*UAS-Pten (II)*) [47]; UAS-Dp110 (*UAS-Pi3K/p110 (III)*) [48]; UAS-DER^DN^ (*UAS DER^DN^*) [49]; UAS-Wee-1 (*UAS-dwee1*) (B. Edgar); UAS-Cyclin E (*UAS-cycE*) (B. Edgar); UAS-Myc (*UAS-Dmyc*) (B. Edgar); UAS-Argos (*UAS-Aos232 III*); Spitz-lacZ (*w; spi[s3547]/CyO*) (Bloomington Stock Center 10462); String-lacZ (*Stringb-E5.3III*) [20] (we screened a battery of transgenic LacZ reporter flies carrying different *cis*-regulatory elements for *string*. Each of these elements partially reproduced the endogenous expression of String: neuroblasts, ectoderm, imaginal discs, etc.). MARCM 40A (*hsFLP, UAS-GFP, FRT40A, tubGAL80; tubGal4/TM6B*) (H. Herranz), MARCM 42D (*hsFLP, UAS-mCD8GFP; FRT42D, tubGAL80; tubGal4*) (*hsFLP, UAS-nGFP, tubGal4; FRT42D, tubGAL80*) (F. Bejarano); MARCM 82B (*hsFLP, UAS-mCD8GFP; tubGal4; tubGAL80, FRT82B*) (N. Perrimon); *RAS1X7B, FRT82B/TM6B* [50]; *SosX122, FRT40A* [51]*; PI3K92Ea, FRT82B/Tm3, ser* [48]; *EGFR1K35, FRT42D/CyO* [52]; *chico/bsk* (*DF(2L)flp147E, FRT40A*) [53,54]; *cycE AR95 E, FRT40A* [21] (B. Edgar); *spi^a14^, FRT40A; hsFLP, FRT40A, ubi-GFP, hsFLP, FRT42D, ubi-GFP.*

### Clonal analysis.

*Twin spot analysis.* To generate twin spot clones in histoblasts, we heat shocked third instar larvae for 1 h at 37 °C.

*Generation of MARCM clones.* The “MARCM ready” flies used for the generation of clones in the histoblasts are listed in the Fly stocks section. Single crosses with the corresponding mutants were sufficient to induce MARCM positively labeled clones [55]. To facilitate in vivo time-lapse analysis of mutant cells, we introduced a general His2-YFP [56] nuclear marker under the control of the ubiquitin promoter allowing mutant cells labeled with green fluorescent protein (GFP) to be compared directly to wild-type cells labeled with His2YFP. For analyzing mutations on the second chromosome, we crossed flies carrying the mutation to flies carrying the His2YFP on the third chromosome. Alternatively, for mutations on the third chromosome, we crossed mutant flies to flies carrying the marker on the second chromosome.

*Generation of MARCM clones: Blastoderm.* Clone induction in the blastoderm was used to generate clones in both histoblasts and LECs. Virgins of the “MARCM ready” stock were crossed en masse to males carrying mutant FRT chromosomes plus or minus a corresponding His2-YFP reporter (see above). We collected eggs on agar plates supplied with yeast. Eggs were collected for 2 h at 25 °C, then allowed to develop for three additional hours at 25 °C, and were finally heat shocked by immersion in a water bath for 1 h at 37 °C. The embryos developed on the agar plates. When the animals reached the second and third instar larval stages, larvae were collected and screened under a GFP dissecting microscope.

*Generation of MARCM clones: Third instar larva.* Alternatively, MARCM clones were induced by heat shocking third instar larvae, which induces clones in histoblasts arrested in G2 (after DNA replication), but not in LECs, as they are terminally differentiated. This procedure will yield observable recombination only after the first cell division, upon pupariation, when one of the daughter cells will become homozygous mutant.

*Clonal overexpression in histoblasts.* For clonal overexpression of UAS constructs, we used a combination of the FLP/FRT system and the Gal4/UAS system by using an *yw hsp70-flp; Act FRT y^+^ FRT-Gal4 UAS-GFP* strain [57]. To be able to follow mutant clones live, we engineered our stocks with a ubiquitously expressed nuclear His2-YFP marker [56] by generating flies of the genotype *yw hsp70-flp; Act FRT y^+^ FRT Gal4 UAS-GFP; His2YFP/TM2* or *His2RFP/TM2*. The proportion of cells that will undergo recombination depends on the severity of the heat shock. Typically, to generate clones in the histoblasts, we heat shocked third instar larvae for 7 min at 37–38 °C.

*Expression of UAS- constructs in LECs.* Expression of UAS constructs in LECs was performed as described previously [58].

### Immunohistochemistry.

Primary antibodies used were mouse anti-dp-ERK (Sigma; 1:1,000), mouse anti-Disc large (Hybridoma Bank; 1:100), mouse anti-βGal (Sigma; 1:500), rabbit anti-βGal (1:1,000; Cappel), and mouse anti-Cyclin E (1:10; H. Richardson).

Secondary antibodies were anti-mouse or anti-rabbit FITC, Cy3, or Cy5 conjugated (Molecular Probes) used at 1:250 dilutions. Immunohistochemistry was performed using standard procedures. For pupal staging, white pupae (0 h APF) were used as reference. The white prepupa were transferred to fresh vials and kept at 25 °C or 29 °C and standard humidity up to disection. Whole pupae were bisected along the anterioposterior axis in sterilized 1× PBS (pH 7.4). The epidermis was detached from the pupal case using forceps and transferred to an Eppendorff tube on ice. Fixation was performed for 10 or 15 min in 4% paraformaldehyde. After fixation, the epidermis was rinsed three times in 1× PBS and permeabilized in sterilized PBT (0.3% Triton in 1× PBS) (3 × 15 min). After permeabilization, the tissue was blocked for 1 h using PBTB (1% Bovine Serum Albumin [BSA] in PBT). Primary antibodies were incubated overnight at 4 °C with gentle shaking. The epidermis was rinsed in 1× PBS, and washed 3 × 15 min in PBTB. After 1 h blocking in PBTB, the secondary antibody was incubated for 3 h at room temperature. After rinsing in 1× PBS, the tissue was stained using DAPI (1 ng/μl) to mark the nuclei. Finally, the tissue was washed 3 × 15 min in 1× PBS, equilibrated in Vectashield (Vector) and mounted on cover slips. Actin staining using phalloidin alone was performed as above after 10-min fixation and omitting the blocking steps.

### In-situ protocol.

To perform whole-mount in situ hybridization of abdominal epidermal tissue, a *string* digoxigenin-labeled RNA probe was generated from the BDGP cDNA clone LD47579. Whole pupae were bisected in sterilized 1× PBS (pH 7.4) and fixed in 4% paraformaldehyde for 20 min at room temperature. After prehybridization for at least 2 h in hybridization solution (50% formamide, 5× SSC, 100 μg/ml tRNA, 50 μg/ml heparin, and 0.1% Tween 20 in DEPC water) at 55 °C, the epidermis was incubated with the denatured probe overnight at 55 °C. The probe was washed off with warm hybridization solution, and samples were incubated in a 1:2,000 dilution of anti-digoxigenin-AP Fab fragments (1% v/v) (Roche Diagnostics) for 2 h at room temperature. Hybridized RNA signals were detected by incubation with NBT/BCIP substrates, and the stained epidermis was mounted in glycerol.

### Imaging and time-lapse microscopy.

Live imaging of early and late pupae was performed as previously described [58]. Images were captured at 5- or 10-min intervals. Confocal microscopes used were Leica TCS 4D, Leica TCS SP2 AOBS, Leica SP5, or Carl Zeiss LSM510. Initial image analysis was performed with Leica Confocal Software and the Imaris 5D (Bitplane) software. ImageJ (NIH Image) was used for mounting of time-lapse movies in AVI format; Photoshop 7.0 (Adobe Corporation) was used for data processing, and QuickTime Pro for compression.

### Flow cytometry.

To study the effects on cell-cycle phasing of EGFR loss of function in late abdominal histoblasts, we performed flow cytometry assays both in wild-type and EGFR mutant conditions. In order to repress EGFR activity in late pupal stages, we expressed an EGFR^DN^ transgene using a permanent Esg-Gal4 driver. Wild-type flies carried this driver without the transgene. To perform the flow cytometry experiments, whole pupae (15 animals staged at 20 h APF for each condition) were bisected in sterilized 1× PBS (pH 7.4) along the anterioposterior axis using a set of Vannas-Tübingen straight scissors from F.S.T. Histoblasts were positively marked by Esg-Gal4 expression driving UAS-GFP. In order to compare cell-cycle profiles, samples of each condition were prepared and run simultaneously. Under a fluorescent dissecting microscope, the anterior region of each half pupa, containing the head and thorax, was cut off, and tracheae were flushed from the epidemis with 1× PBS using a P200 pipette. The clean epidermis, still attached to the pupal case, was collected in a 12-well culture dish (Nunclon) containing MM3 medium and kept on ice until dissection of all animals was completed. The epidermis was then rinsed three times in 1× PBS to remove the remaining medium and incubated in 9× Trypsin-1× PBS, with 1 mg/ml Hoechst 33342 for 1.5 h at room temperature. Histoblasts were recovered in low-retention Kisker-Biotech tubes, and trypsinization was stopped by adding BSA up to 0.5%. We used a MoFlo flow cytometer (DakoCytomation). Excitation was performed with an argon-ion laser of Coherent Enterprise II and the optical alignment obtained with fluorescent particles of a diameter of 10 μm (Flowcheck; Coulter Corporation). Different populations were defined combining green (GFP) and blue (Hoechst 33342) emissions and the refringency parameters FSC and SSC. Statistic cell-cycle analysis was performed with WinCycle software (Phoenix Flow Systems).

## Supporting Information

Figure S1 Histoblasts Undergo Two Distinct Phases of Proliferation (A and B) Snapshots from a time-lapse movie recording histoblast cell divisions (anterior dorsal nest labeled with an ubiquitously expressed DE-Cadherin-GFP). Selected cells are highlighted in white.
(A) Prior to the onset of proliferation (0 h APF), the apical membranes are highly folded. At 100 min, the histoblasts round up and undergo the first cell cycle, giving rise to two small daughter cells; 140 min later, the two cells have not increased in size. At 260 min, one of the cells divides again to give birth to two even smaller cells. The duration of one cell cycle is about 2.5 h.
(B) During the second stage of proliferation (15 h APF), the length of the cell cycle increases, and cells grow between divisions. The labeled cell divides, and after 5 h, the daughter cells have grown to the size of their mother before they divide again.
(2.30 MB TIF)

Figure S2 Analysis of *son of sevenless* Mutant Clones (A) Snapshots from a time-lapse analysis (Movie S4) of *sos* mutant cells (anterior dorsal nest: 0 h to 8 h APF). A single-mutant GFP-labeled cell was generated in the blastoderm. This cell proliferates at normal rates up to 8 h APF. Histoblasts and LECs were labeled with His2YFP (red).
(B) Twin clonal analysis of *son of sevenless* (*sos*) mutants. Late proliferation defects were observed (two mutant clones with the corresponding twin [yellow lines] in the dorsal nest at 24 h APF).
(2.09 MB TIF)

Figure S3 Growth of Histoblasts during Larval Stages (A) GFP-labeled histoblasts (Esg-Gal4) just after embryo hatching.
(B) GFP-labeled histoblasts at the same magnification from an early third instar larva, showing a dramatic increase in cell size during larval stages.
(2.92 MB TIF)

Movie S1 The Early Cell Cycles of Histoblasts Are Fast and Synchronous and Lead to a Reduction in Cell Size (1–5 h APF) A single histoblast (arrow) in the anterior dorsal nest was labeled with GFP using the FLP-OUT/FRT system. The rest of the cells in the nest were marked using a nuclear His2-YFP (red). The GFP-positive cell divides with a cell doubling time of 2.5 h, generating a clone composed of four smaller cells.
(2.14 MB MOV)

Movie S2 In the Second Proliferation Stage, Histoblasts Grow and Maintain a Constant Cell Size as the Cell Cycle Slows Down and Divisions Become Asynchronous (16–24 h APF) Cells were labeled using a ubiquitously expressed DE-Cadherin GFP fusion protein. A single cell in the posterior dorsal nest undergoing mitosis was outlined in white and its daughter cells in red and blue. After the first division, the two daughter cells do not divide for 5 h but grow to the size of the mother cell. Subsequently, one of the daughter cells divides again. During this stage, cell divisions occurred stochastically (observe the random distribution of big round cells entering mitosis).
(6.23 MB MOV)

Movie S3 EGFR Signaling Is Not Required for Histoblast Proliferation during the Early Cell Cycles (1–8 h APF) Expression of dominant-negative EGFR protein in histoblasts (ventral nest expressing UAS-GFP and UAS-DER^DN^ under the control of the permanent Esg-Gal4 driver) does not affect early cell divisions, and histoblasts undergo three synchronous cell cycles by 8 h APF.
(2.21 MB MOV)

Movie S4 Ras Signaling Is Not Required for Histoblast Proliferation during the Early Cell Cycles (1–8 h APF) A single mutant cell for the Ras-GEF *son of sevenless* (*sos*) (anterior dorsal nest) was induced in the blastoderm by mitotic recombination and labeled with GFP (green) using the MARCM system. Wild-type cells were labeled by expression of a nuclear His2-YFP (red). The mutant cell initiates the early cell cycles in synchrony with the wild-type histoblasts and divides three times to form a clone composed of eight cells at 8 h APF. Note that the third division (as the mutant cell approaches the transition to the second stage of proliferation) is slightly delayed compared to wild-type neighbors.
(6.80 MB MOV)

Movie S5 PI3K Activity Is Not Required during the Early Cell Divisions Stage (1–8 h APF) A homozygous mutant cell for *dp110* in the anterior dorsal nest was induced in the blastoderm by mitotic recombination and labeled with GFP (green) using the MARCM system. Wild-type cells were labeled by expression of a nuclear His2-YFP (red). The mutant cell undergoes the first cell cycles at normal rates and forms a clone composed of eight cells. Arrows point the synchronous entry in mitosis of the *dp110* mutant cell and its wild-type neighbor.
(4.99 MB MOV)

Movie S6 PI3K Signaling Is Necessary for Cell-Cycle Progression during the Second Stage of Proliferation (18–27 h APF) Homozygous mutant clones for *dp110* induced in the blastoderm (MARCM, labeled with GFP) and monitored during the second stage of histoblast proliferation (posterior dorsal nest). Wild-type cells were labeled by expression of a nuclear His2-YFP (red). At 18 h APF, a clone from a single precursor histoblast should include around 20 cells generated by three synchronous fast early divisions and one or two slow late divisions. The clone in the movie is initially composed of eight cells, indicating that the mutant histoblasts are already delayed in their entry in the slow stage and did not proceed through a fourth division yet. The entry in division of mutant histoblasts was compared to neighbor wild-type cells (dots). The histoblasts from the mutant clone enter division at a very slow pace (compared to wild-type neighbors) and some cells do not divide at all. In total, the clone reaches a size of 14 cells by 27 h APF.
(3.79 MB MOV)

Movie S7 Inhibition of PI3K Activity Does Not Affect the Speed of the Early Cell Cycles (1–8 h APF) Histoblasts (anterior dorsal nest) expressing UAS-PTEN undergo the first cell cycles at normal speed. Clones were induced using the hsFLP/FRT system by heat shock in an early third instar larva and marked using GFP (green; arrows). The nuclei of all cells were labeled with ubiquitously expressed His2-YFP.
(3.17 MB MOV)

## Abbreviations

APF: after puparium formation
EGFR: epidermal growth factor receptor
LEC: larval epidermal cell
PI3K: phosphoinositide 3-kinase
