# Supplementary figures and images for: Dynamic Control of Cell Cycle and Growth Coupling by Ecdysone, EGFR, and PI3K Signaling in Drosophila Histoblasts
Source: PLoS Biol. 2009 Apr 7;7(4):e1000079. doi: 10.1371/journal.pbio.1000079 (PMC2672598; doi:10.1371/journal.pbio.1000079)

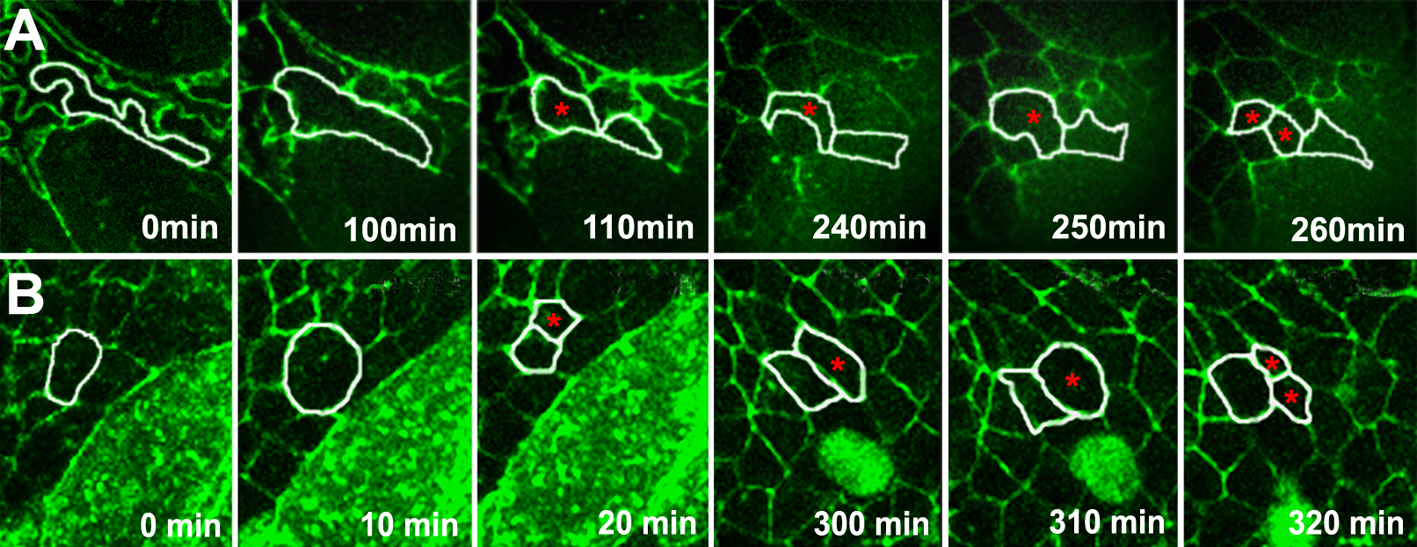

Supplement: Figure S1 — (A and B) Snapshots from a time-lapse movie recording histoblast cell divisions (anterior dorsal nest labeled with an ubiquitously expressed DE-Cadherin-GFP). Selected cells are highlighted in white. (A) Prior to the onset of proliferation (0 h APF), the apical membranes are highly folded. At 100 min, the histoblasts round up and undergo the first cell cycle, giving rise to two small daughter cells; 140 min later, the two cells have not increased in size. At 260 min, one of the cells divides again to give birth to two even smaller cells. The duration of one cell cycle is about 2.5 h. (B) During the second stage of proliferation (15 h APF), the length of the cell cycle increases, and cells grow between divisions. The labeled cell divides, and after 5 h, the daughter cells have grown to the size of their mother before they divide again. (2.30 MB TIF) [file pbio.1000079.sg001.tif]

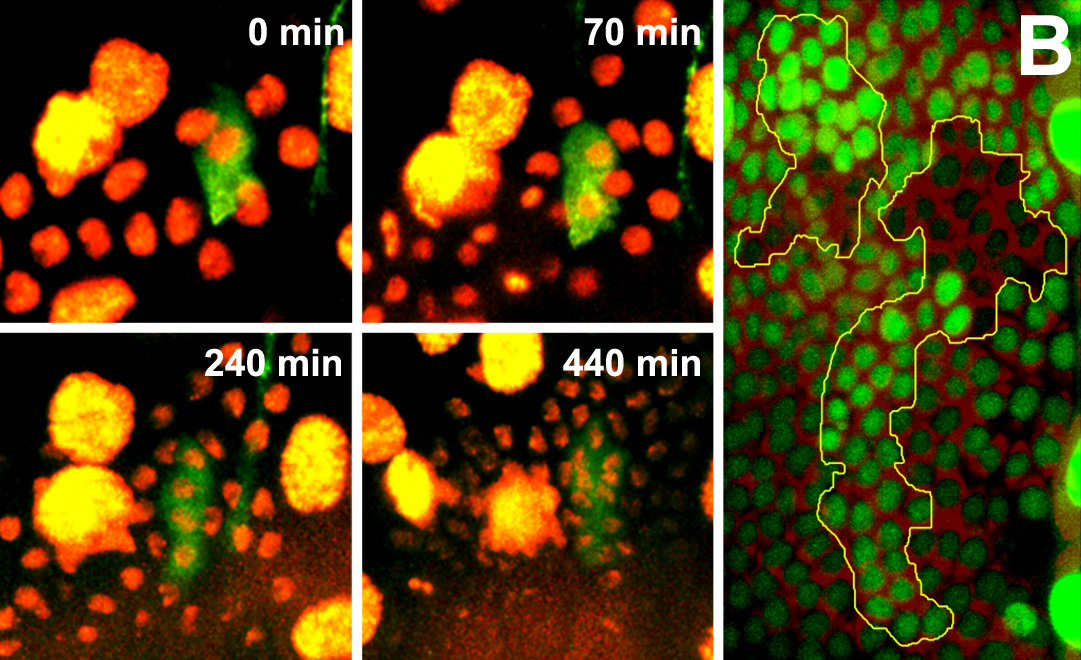

Supplement: Figure S2 — (A) Snapshots from a time-lapse analysis (Movie S4) of sos mutant cells (anterior dorsal nest: 0 h to 8 h APF). A single-mutant GFP-labeled cell was generated in the blastoderm. This cell proliferates at normal rates up to 8 h APF. Histoblasts and LECs were labeled with His2YFP (red). (B) Twin clonal analysis of son of sevenless (sos) mutants. Late proliferation defects were observed (two mutant clones with the corresponding twin [yellow lines] in the dorsal nest at 24 h APF). (2.09 MB TIF) [file pbio.1000079.sg002.tif]

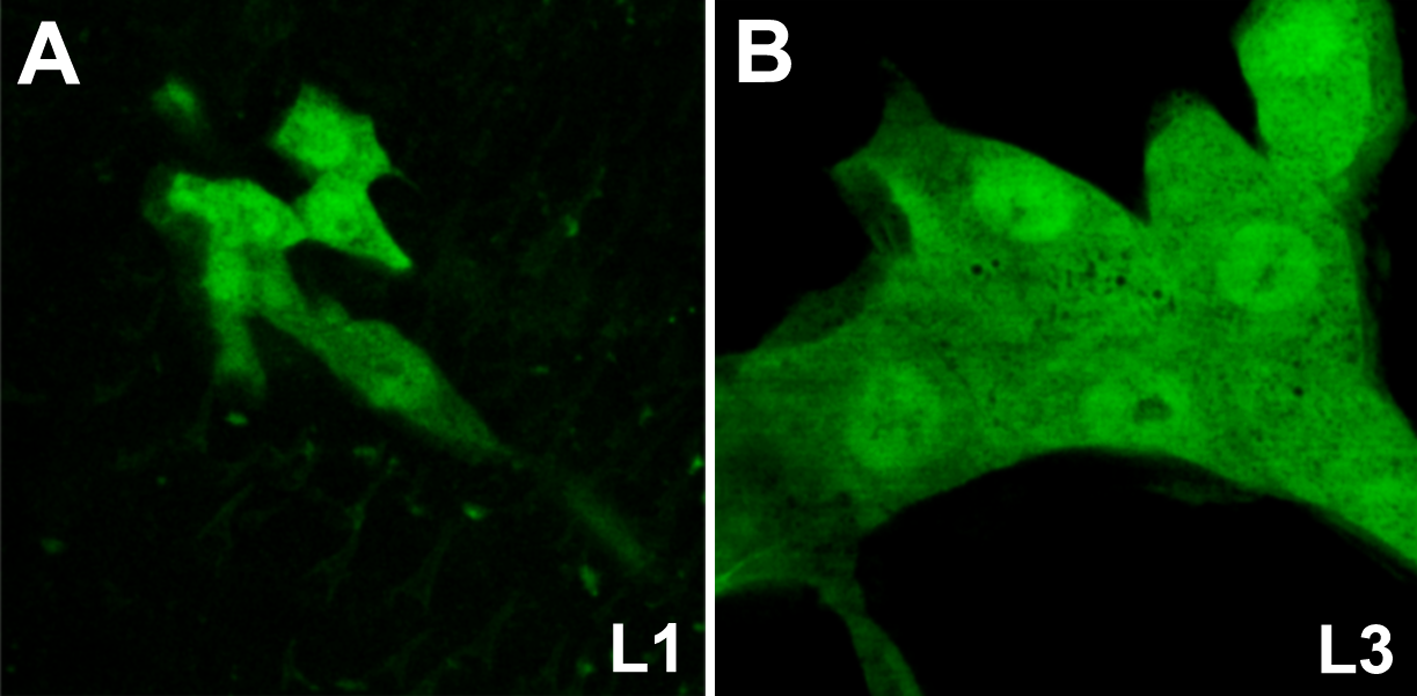

Supplement: Figure S3 — (A) GFP-labeled histoblasts (Esg-Gal4) just after embryo hatching. (B) GFP-labeled histoblasts at the same magnification from an early third instar larva, showing a dramatic increase in cell size during larval stages. (2.92 MB TIF) [file pbio.1000079.sg003.tif]
